# Polymyxin Stereochemistry and Its Role in Antibacterial Activity and Outer Membrane Disruption

**DOI:** 10.1021/acsinfecdis.2c00307

**Published:** 2022-11-07

**Authors:** Cornelis J. Slingerland, Ioli Kotsogianni, Charlotte M. J. Wesseling, Nathaniel I. Martin

**Affiliations:** Biological Chemistry Group, Institute of Biology Leiden, Leiden University, Sylviusweg 72, 2333 BE Leiden, The Netherlands

**Keywords:** polymyxin B, polymyxin B nonapeptide, mechanism of action, stereochemistry, enantiomers

## Abstract

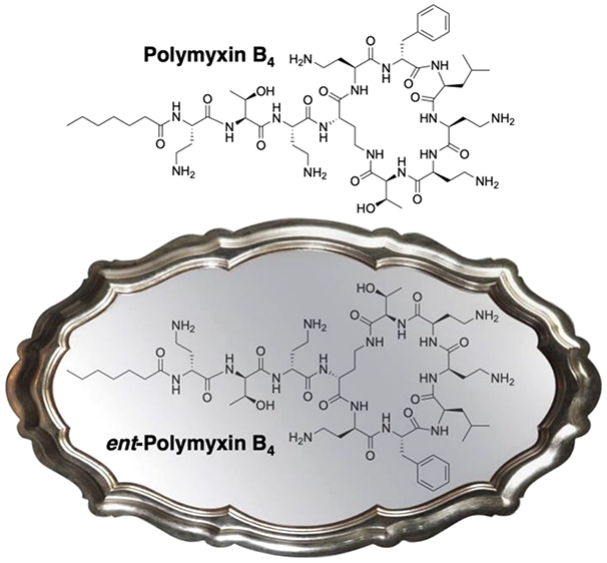

With increasing rates of resistance toward commonly used antibiotics, especially among Gram-negative bacteria, there is renewed interested in polymyxins. Polymyxins are lipopeptide antibiotics with potent anti-Gram-negative activity and are generally believed to target lipid A, the lipopolysaccharide (LPS) anchor found in the outer membrane of Gram-negative bacteria. To characterize the stereochemical aspects of their mechanism(s) of action, we synthesized the full enantiomers of polymyxin B and the polymyxin B nonapeptide (PMBN). Both compounds were compared with the natural compounds in biological and biophysical assays, revealing strongly reduced antibacterial activity for the enantiomeric species. The enantiomeric compounds also exhibit reduced LPS binding, lower outer membrane (OM) permeabilization, and loss of synergetic potential. These findings provide new insights into the stereochemical requirements underlying the mechanisms of action of polymyxin B and PMBN.

Polymyxin antibiotics are a family of cyclic lipopeptides among which polymyxin B and polymyxin E (colistin) are used clinically (Figure 1A). Given their potent and specific anti-Gram-negative activity, the clinical application of polymyxins is on the rise as a result of global dissemination of multidrug resistant bacteria.^1,2^ Produced by the soil dwelling microorganism *Paenibacillus polymyxa*,^3,4^ polymyxins comprise a class of polycationic decapeptides carrying a lipophilic tail. Due to the presence of several nonproteogenic diaminobutyric acid (Dab) residues, the peptide is polycationic at physiological pH. The decapeptide part consists of a seven-membered ring with three exocyclic residues connecting the ring to the N-terminal lipophilic tail.^5^ Polymyxins are typically obtained and clinically used as a mixture of closely related congeners.^2^ While polymyxins B and E differ with respect to the amino acid at position 6 (d-Phe and d-Leu, respectively), minor variations are also found in the lipophilic acyl tail, as exemplified by the structures of polymyxins B_1_ and B_4_ (Figure 1A).^5^ Previous studies into polymyxin B and polymyxin E variants bearing slightly different lipid tails have shown them to display similar antibacterial activities.^6^

**Figure 1:**
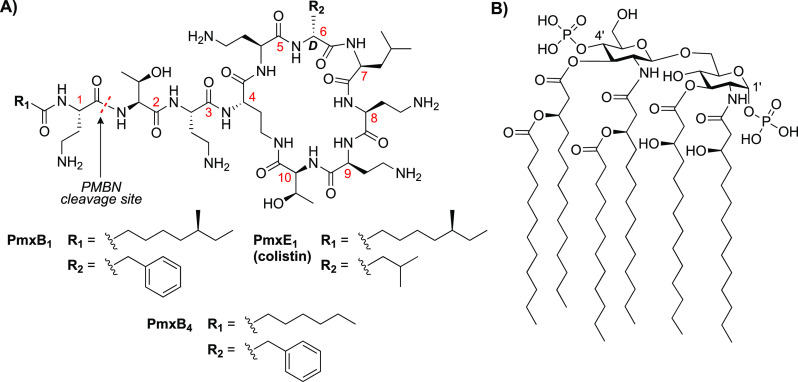
Polymyxins and their target lipid A. (A) Structures of clinically used polymyxin B and polymyxin E (colistin) including the numbering typically used for polymyxin residues. (B) Structure of lipid A (from *E. coli* K-12), the LPS membrane anchoring lipid in Gram-negative bacteria.

The primary mechanism by which the polymyxins target Gram-negative bacteria is by binding to lipid A,^7−9^ the membrane anchoring moiety of the lipopolysaccharide (LPS) comprising the outer membrane (OM) of Gram-negative cells. Lipid A is a structurally complex biomolecule composed of a disaccharide consisting of two phosphorylated glucosamine units that are decorated with a number of acyl tails that facilitate embedding in the OM (Figure 1B). Polymyxin sensitivity and resistance is strongly dependent on the structural features of lipid A. Notably, many modified lipid A variants exist naturally, either in a species specific fashion or as the result of acquired resistance genes. For instance, *Neisseria gonorrhoeae* is intrinsically resistant to polymyxins as a result of the inclusion of a phosophoethanolamine moiety at the 4′ position of the lipid A headgroup, which serves to reduce its binding by polymyxins.^10,11^ A similar lipid A modification is observed as a result of the recently reported plasmid-mediated, mobile colistin resistance (mcr) genes, which encode for phosphoethanolamine transferases that also modify the structure of lipid A at the 1′ position and in doing so confer polymyxin resistance.^12−14^

From a mechanistic perspective, the activity of the cationic polymyxins is ascribed to their electrostatic interactions with the negatively charged phosphate groups of lipid A, which leads to displacement of divalent cations that bridge adjacent phosphate containing molecules.^2,9,15−17^ As a result, “OM loosening” occurs, which in turn allows for polymyxin passage across the OM.^17,18^ With access to the periplasmic space, the polymyxins go on to exert their secondary effects on the cytoplasmic membrane.^4,13^ Interestingly, recent studies have also suggested that the bactericidal effect of the polymyxins is mediated by their engagement with nascent LPS present in the cytoplasmic membrane.^19^

It is also well established that the nonapeptide fragment obtained by enzymatic degradation of polymyxin B (Figure 1A) retains the OM disrupting abilities of the parent compound despite lacking antibiotic activity. This finding has spurred a number of investigations into the use of polymyxin B nonapeptide (PMBN) as a synergist capable of potentiating the activity of antibiotics that typically cannot cross the Gram-negative OM.^20−22^ In addition, several studies describing the development of synthetic PMBN analogues as antibiotic adjuvants have been reported in recent years.^23−25^

Structure–activity relationship studies aimed at elucidating the contribution of component amino acid stereochemistry to the activity of the polymyxins and PMBN have also been reported.^26,27^ In a recent study, the Reymond group generated a library of stereorandomized polymyxin B analogues that in some cases exhibited good-to-moderate activity against *E. coli*.^26^ In addition, Fridkin and co-workers previously prepared and characterized the activity of the enantiomeric form of PMBN, showing it to be a much less active antibiotic synergist.^27^ Absent from these prior studies, however, is an assessment of the completely mirror-image enantiomeric form of any full-length polymyxin. Such mirror-image strategies have been previously applied by our group and others to better understand the stereochemical parameters governing the mechanisms of various peptide antibiotics.^28−32^ In those cases where an achiral target is implicated, the enantiomeric form of the peptide antibiotic generally exhibits activity on par with the natural product. This is the case for laspartomycin, which targets undecaprenyl phosphate (C_55_-P)^32^ as well as the clinically used bacitracin, which targets undecaprenyl pyrophosphate (C_55_-PP).^31^ In contrast, for peptide antibiotics with an implicated chiral biomolecular target, the activity of the corresponding enantiomer is often significantly reduced. Specific examples include the recently reported laterocidine (targeting lipid A),^30^ tridecaptin A1 (targeting the bacterial cell wall precursor lipid II),^29^ thanatin (targeting LptA and LptD proteins involved in LPS biosynthesis),^28^ and daptomycin (targeting phosphatidylglycerol).^33^ In all of these examples, the antibacterial activity of the corresponding enantiomers was shown to be either severely decreased or abolished. Using a similar approach, we here describe the synthesis of enantiomeric polymyxin B4 along with the corresponding PMBN enantiomer. The activities of the enantiomeric species were subsequently compared to their natural counterparts in a variety of biological and biophysical assays, providing new insights into the stereochemical requirements that govern the antibacterial mechanism of action of the polymyxins.

In considering which polymyxin to use for mirror image analysis, we selected polymyxin B_4_ (PmxB_4_, Figure 1A). Bearing a linear C7 lipid, PmxB_4_ has been previously shown to have the same activity as polymyxin B_1_^6^ with the C7 lipid offering the advantage of having one less stereogenic center to be concerned with when synthesizing the enantiomeric species. To date, a number of solid phase syntheses of polymyxin B have been reported.^6,34,35^ Inspired by these, both *ent*-PmxB_4_ and *ent*-PMBN were synthesized using a combined solid- and solution-phase approach (Scheme 1). The protected linear peptide intermediates were assembled on a solid support using 2-chlorotrityl chloro (CTC) resin and the corresponding enantiomeric Fmoc amino acid building blocks starting from d-Thr10. As indicated in Scheme 1, the linear *ent*-PmxB_4_ precursor was assembled to include the N-terminal Dab residue bearing the heptanoic acid moiety, while the *ent*-PMBN precursor peptide terminated with Boc-Thr. In both cases, an orthogonal protecting group strategy was required for the amino side chain of the d-Dab residue at position 4 given its role in the subsequent ring closure. While previous reports have described the use of Alloc^35^ or Mtt^34^ protecting groups for this purpose, we found that using the corresponding azide was more practical. To this end, we incorporated Fmoc-d-Dab(N_3_) at position 4 in the linear peptide intermediates. Once the linear peptides were assembled, the azide moiety of d-Dab(N_3_) was cleanly reduced by treatment with DTT/DIPA in dry DMF.^36^ After the reduction was deemed complete by LC/MS analysis, the protected peptides were cleaved from the resin by HFIP treatment and cyclized in solution. Using this strategy, both *ent*-PmxB_4_ and PmxB_4_, as well as *ent*-PMBN, were prepared using the appropriate d- or l-Fmoc-amino acids. In the case of PMBN with natural stereochemistry, we elected to use the well-established route wherein the parent natural product is enzymatically degraded using ficin.^37,38^ HPLC analysis and HRMS analysis (Table S1, Figures S1 and S2) confirmed the identity and expected equivalence of *ent*-PmxB_4_ to PmxB_4_ and *ent*-PMBN to PMBN and *ent*-PMBN. In addition and as expected, the obtained high field NMR spectra (^1^H and 2D NOESY) were found to be identical for the enantiomeric pairs (Figures S3–S6).

**Scheme 1:**
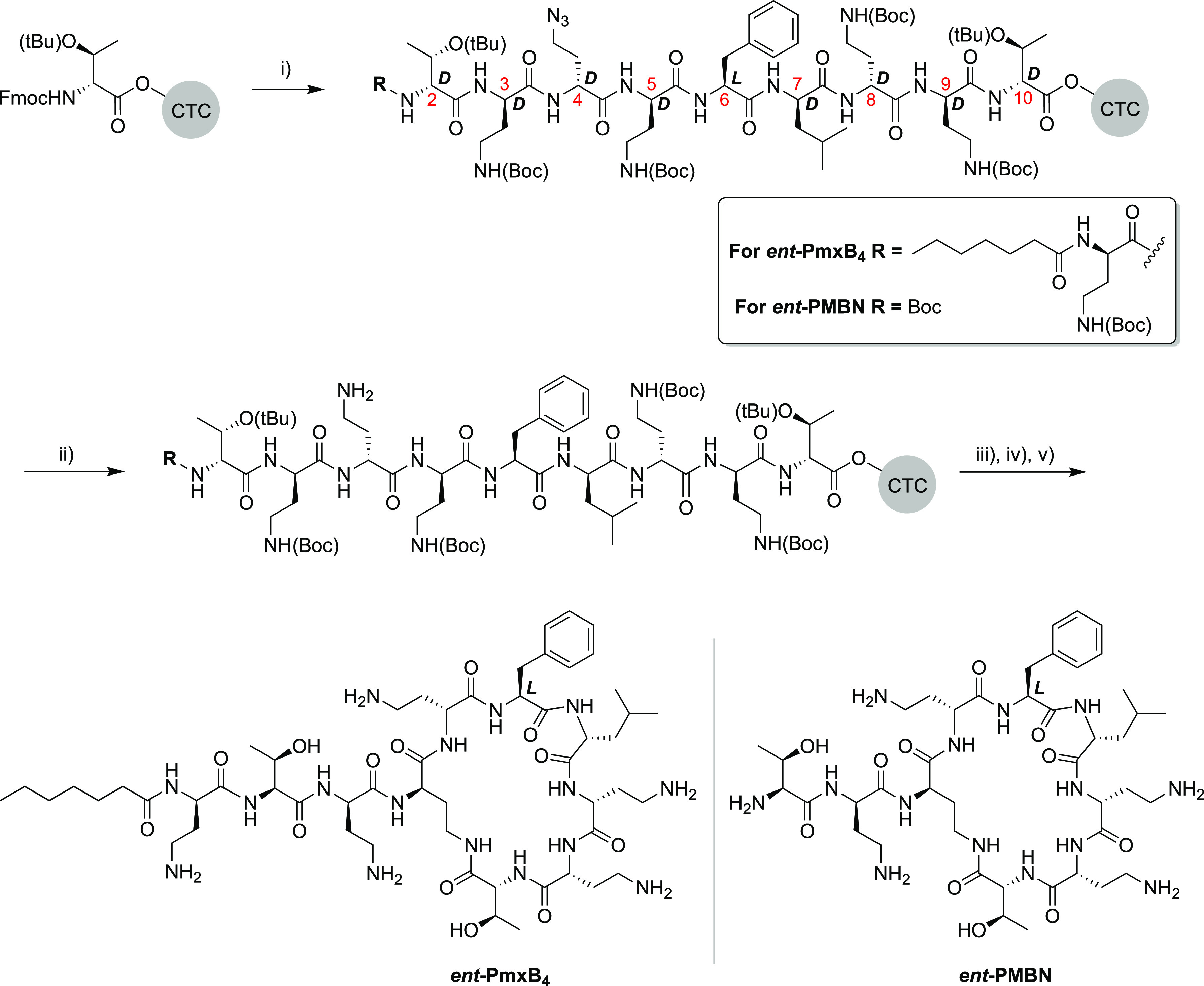
Solid Phase Peptide Synthesis (SPPS) of Enantiomeric Polymyxin B4 (*ent*-PmxB4) and Enantiomeric Polymyxin B Nonapeptide (*ent*-PMBN) Reagents and conditions: (i) standard SPPS with 4 equiv of FmocAA (coupling: BOP/DIPEA, deprotection: 20% piperidine in DMF) to yield the resin-bound linear peptide intermediates; (ii) on-resin azide group reduction with DTT, DIPA, dry DMF, RT, 3 × 2 h; (iii) mild acid resin cleavage with HFIP/DCM (20/80), 1.5 h; (iv) solution-phase cyclization with DIC/Oxyma, DMF, o/n; (v) global deprotection with TFA, TIPS, H_2_O.

We next evaluated the activity of both PmxB_4_ and *ent*-PmxB_4_ against several strains of Gram-negative bacteria (Tables 1 and S2). While PmxB_4_ was found to exhibit low minimal inhibitory concentration (MIC) values in line with the clinically used polymyxin B, this was clearly not the case for *ent*-PmxB_4_ as the enantiomeric species was found to be devoid of activity. Only at the highest concentration tested of 128 μg/mL was the growth of the *E. coli* strains impacted, while for *K. pneumoniae*, *A. baumannii*, and *P. aeruginosa*, no activity was observed. The addition of known OM disrupting agents like PMBN or the PMBN derivative SPR741^39^ also did not improve the activity of *ent*-PmxB_4_ (Table S3). Also of note, while *E. coli* strains with severely truncated LPS (as in the ΔwaaD and ΔwaaC mutants) were found to have increased sensitivity to PmxB_4_, the activity of *ent*-PmxB4 was not enhanced (Table 1). These results show that the full LPS structure is not required for the action of PmxB_4_, a finding supportive of the recent suggestion that the core sugars of LPS might in fact lower the efficacy of polymyxin antibiotics by hindering them from reaching lipid A.^40^ In contrast, the absence of a complete LPS layer did not serve to enhance the activity of *ent*-PmxB_4_, further demonstrating the high degree of stereospecificity that underpins the antibacterial mechanism of the polymyxins.

**Table 1:** Minimal Inhibitory Concentration (MIC) Values for PmxB4 and *ent*-PmxB4^a^

	strain	PmxB_4_	*ent*-PmxB_4_	PmxB (commercial)
*E. coli*	ATCC 25922	0.25	128	1
	BW25113	0.5	>128	1
	*mcr*-1	4	128	4
	JW3594 (ΔwaaD)	0.06	128	0.06
	JW3596 (ΔwaaC)	0.06	128	0.06
*K. pneumoniae*	ATCC 13883	0.125	>128	0.25
*A. baumannii*	ATCC 19606	0.5	>128	0.25
*P. aeruginosa*	ATCC 27853	0.25	>128	1
	NRZ03961	0.25	>128	1
	ATCC 10145	0.25	>128	1
	2018-007	0.5	>128	2

a MIC values are derived from triplicate experiments and are expressed as μg/mL. See Figure S7 for the analysis of PmxB obtained from commercial sources.

Having established that *ent*-PmxB4 is devoid of inherent antibacterial activity, we next turned our attention to evaluating its potential as an antibiotic synergist. The rationale for doing so was inspired by a recent report from Brown and co-workers who found that, when tested against polymyxin-resistant (*mcr*-positive) strains, polymyxins exhibit synergistic activity with Gram-positive specific antibiotics like rifampicin.^41^ This is notable in that it suggests that, while strong lipid A binding is needed for antibacterial activity, it is not a prerequisite for synergistic activity. Also of note is another study, again from the Brown group, which revealed that PMBN loses its synergistic activity against *mcr*-positive strains, implying that the lipid tail in full-length polymyxins is needed to maintain synergy against polymyxin-resistant bacteria.^42^ To this end, we performed a series of checkerboard assays to assess the capacity of the enantiomeric pairs PmxB_4_ and *ent*-PmxB_4_ as well as PMBN and *ent*-PMBN to potentiate the activity of rifampicin against both polymyxin-sensitive and polymyxin-resistant strains (Figure 2).

**Figure 2:**
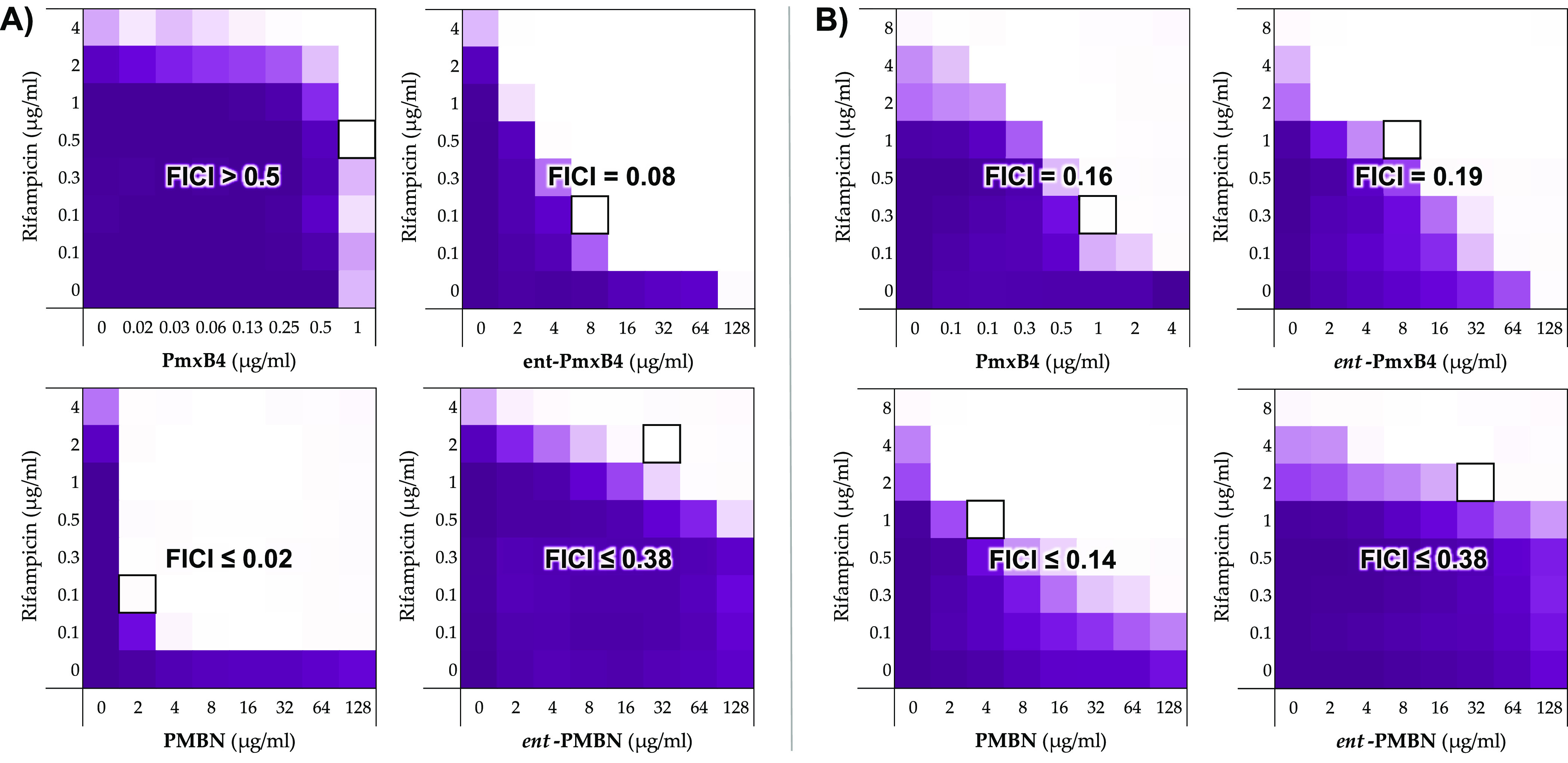
Analysis of rifampicin potentiation by enantiomeric pairs PmxB_4_/*ent*-PmxB_4_ and PMBN/*ent*-PMBN against polymyxin-sensitive and polymyxin-resistant bacteria. (A) Checkerboard assays to assess the synergy of rifampicin with PmxB4/*ent*-PmxB4 and PMBN/*ent*-PMBN against polymyxin-sensitive *E. coli* ATCC 25922. (B) Checkerboard assays to assess the synergy of rifampicin with PmxB4/*ent*-PmxB4 and PMBN/*ent*-PMBN against polymyxin-resistant (*mcr*-1 positive) *E. coli*. Color corresponds to growth (as read by OD_600_) after incubation and with intensity proportional to the observed OD_600_ value. White areas indicate no growth. Bounded box indicates the combination of concentrations that gives the lowest FIC index value. Values used for FICI calculations are shown in Table S4.

Given the potent inherent antimicrobial activity of polymyxin antibiotics, they are not typically examined for synergism with other antibiotics. In keeping with this and as illustrated in Figure 2A, PmxB_4_ is not an effective synergist of rifampicin against polymyxin-sensitive *E. coli* due largely to its already low MIC (2 μg/mL) against the strain used. In contrast, *ent*-pmxB_4_ shows a very clear synergistic effect with rifampicin. Specifically, while *ent*-PmxB_4_ alone has a very high MIC of 128 μg/mL, when administered at 8 μg/mL, it very effectively lowers the MIC of rifampicin from 8 to 0.1 μg/mL, corresponding to a calculated FICI value of 0.075. In contrast, when comparing the synergistic activity of PMBN and *ent*-PMBN, the opposite trend is observed. It is well established that, despite lacking the inherent antimicrobial activity of the parent lipopeptide, PMBN is a potent synergist of many antibiotics, including rifampicin, that are generally only used to treat Gram-positive infections.^43,24^ In this regard and as expected, our assessment of the effect of PMBN on the activity of rifampicin against polymyxin-sensitive *E. coli* revealed a strong synergistic effect with a corresponding calculated FICI value of 0.020. In comparison and as readily seen from the checkerboard data, only a minor potentiation of rifampicin is achieved by *ent*-PMBN, requiring high concentrations and corresponding to an FICI value of 0.375. These findings are in agreement with the previous studies of Fridkin and co-workers who found *ent*-PMBN to also be a poor potentiator of novobiocin and erythromycin.^27^ We next examined the synergistic activity of rifampicin with the PmxB_4_/*ent*-PmxB_4_ and PMBN/*ent*-PMBN enantiomeric pairs in the context of an *mcr*-1 positive *E. coli* strain (Figure 2B). Of particular note is the finding that in this case PmxB_4_ and *ent*-PmxB_4_ exhibit a similarly moderate degree of synergy with FICI values of 0.163 and 0.188, respectively. In the case of PMBN, the synergistic activity is heavily impacted compared to the polymyxin-sensitive strain, resulting in an increased FICI value of 0.141, while for *ent*-PMBN, the same low level of synergy was found (FICI 0.375). Taken together, these findings support the idea that, in the absence of specific lipid A binding, an effective synergist likely needs to be both polycationic in nature and include a membrane-anchoring hydrophobic moiety.

We next investigated the role of OM perturbation in the observed potentiation of rifampicin by PmxB_4_/*ent*-PmxB_4_ and PMBN/*ent*-PMBN. To do so, we applied a well-established fluorescence-based assay that reports the uptake of *N*-phenyl-1-naphthylamine (NPN). Only upon OM disruption can the hydrophobic NPN gain access to the phospholipid layer, resulting in an increase in fluorescence.^44^ Previous studies have validated this assay for assessing the relative activity of OM disrupting agents, most notably PMBN, which causes a clear increase in fluorescence when added to Gram-negative bacteria in the presence of NPN.^16,22^ For our investigations, we characterized the OM perturbation of three different *E. coli* strains: two polymyxin-sensitive strains containing either smooth LPS (ATCC 25922) or rough LPS (BW25113) (Figure 3A,B) and one polymyxin-resistant strain containing phosphoethanolamine modified LPS (*mcr*-1 positive) (Figure 3C).

**Figure 3:**
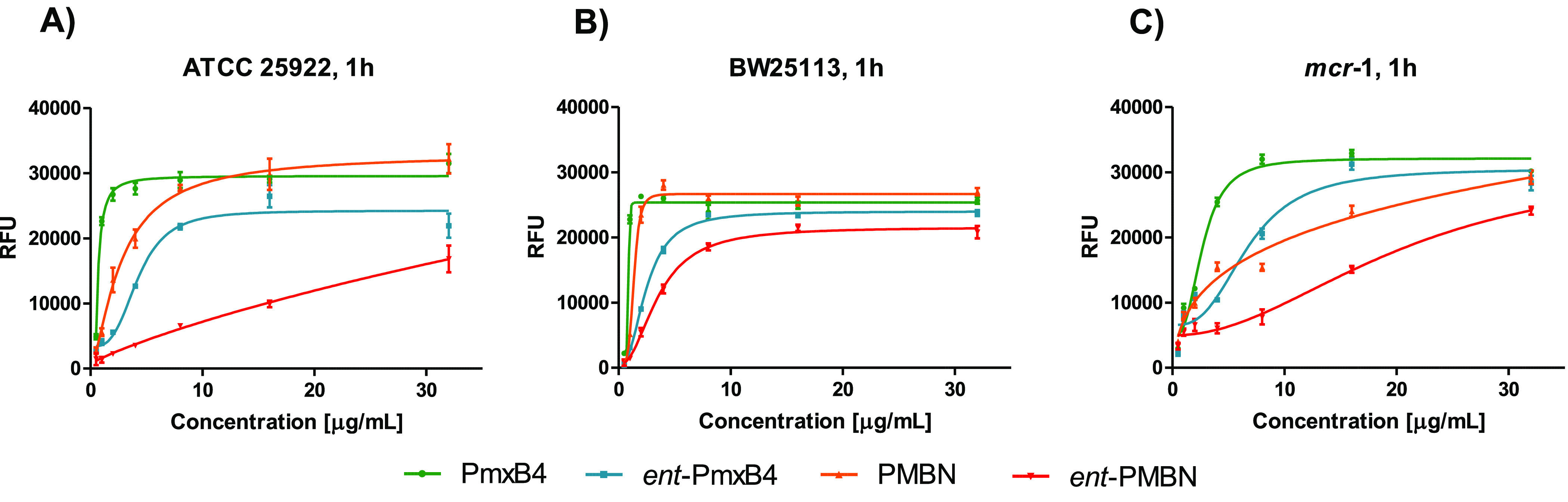
*N*-Phenyl-1-naphthyl (NPN) uptake in *E. coli* upon incubation with PmxB_4_/ent-PmxB_4_ and PMBN/ent-PMBN. (A, B) NPN uptake in polymyxin-sensitive strains ATCC 25922 (smooth LPS) and BW25113 (rough LPS); (C) NPN uptake in the polymyxin-resistant, *mcr*-1 positive strain. Values and associated error bars are based on triplicates with fluorescence read-out after 1 h of incubation. RFU: relative fluorescence unit.

As seen in Figure 3A,B, against the polymyxin-sensitive *E. coli* strains tested, both PmxB_4_ and PMBN were found to elicit potent OM disruption with PMBN acting more effectively on the rough LPS strain BW21153. In comparison, *ent*-PmxB_4_ was found to induce moderate OM disruption against both polymyxin-sensitive strains. Interestingly, while *ent*-PMBN showed only weak OM disruption against the *E. coli* strain ATCC 25922 containing smooth LPS, against the BW25113 strain (containing rough LPS), a higher degree of OM perturbation was observed. These findings indicate that in general the rough LPS containing BW25113 strain appears to have a greater sensitivity to OM disruption based on the NPN assay. In line with the results of the synergy studies, against the *mcr*-positive *E. coli* strain, higher concentrations of all four compounds were required to achieve NPN uptake (Figure 3C). In this case, PmxB_4_ elicits the most effective OM disruption with *ent*-PmxB_4_ also achieving full NPN uptake albeit at higher concentrations. In comparison, both PMBN and *ent*-PmxB4 failed to achieve full NPN uptake at the highest concentration tested of 32 μg/mL. Taken together, these data again support the conclusion that, in developing OM disrupting agents with activity against strains bearing structurally modified lipid A, the presence of a lipophilic moiety is essential.

We also used isothermal titration calorimetry (ITC) to study target engagement by PmxB_4_/*ent*-PmxB_4_ and PMBN/*ent*-PMBN (Figure 4). Previous reports using ITC to characterize the interaction of polymyxins with LPS have revealed it to be a complex process that is highly dependent on the source of the LPS and the experimental conditions used.^9,45−47^ For our investigations, we elected to use a commercially available LPS preparation (derived from *E. coli* O55:B5) that has also been used previously for ITC studies aimed at assessing binding interactions with polymyxins.^45^ We found that titration of the polymyxin analogues (1 mM solution) into the sample cell containing LPS (at an estimated concentration of 20 μM) gave the most reproducible results. An exothermic interaction was observed upon titration of PmxB_4_ into the LPS solution (Figure 4A), while titration of PmxB_4_ into buffer alone yielded no signal (Figure S9), indicating that the heat measured derives from interactions with LPS. In agreement with previous reports, the thermograms resulting from the titration of PmxB_4_ into the LPS solution are complex and do not fit a one-step binding process.^45,46^ For this reason, a simple dissociation constant cannot be reliably determined for the interaction. In addition, because the LPS used is a complex mixture, an estimate of its molecular weight (20 kDa) was applied, which in turn precludes a precise determination of the molar ratios for the binding of LPS by the polymyxins here studied. It is, however, possible to compare the relative enthalpies of LPS binding for the different polymyxins along with the shapes of the resulting binding thermograms. The thermogram obtained upon titration of *ent*-PmxB_4_ into LPS shows a different pattern than that observed for the titration with PmxB_4_ (Figure 4A, upper right). While an exothermic interaction was still observed, the corresponding Δ*H* is much smaller (Figure 4B), indicating a less productive interaction with LPS. In addition, saturation took longer to achieve, suggesting that more equivalents are needed to occupy the available binding sites on the LPS. Interestingly, for PMBN and *ent*-PMBN, the thermograms are quite similar, both showing saturation at about 70 min (Figure 4A bottom) with a slightly smaller Δ*H* measured for the enantiomeric species. These data suggest that, despite PMBN being a vastly superior OM disrupting agent and antibiotic synergist compared to *ent*-PMBN, both appear to interact with LPS in a similar manner. Notably, this finding is in agreement with previous studies where PMBN and *ent*-PMBN were found to have comparable LPS interactions based on their capacities to similarly displace dansyl-labeled PMBN from intact *E. coli*.^27^

**Figure 4:**
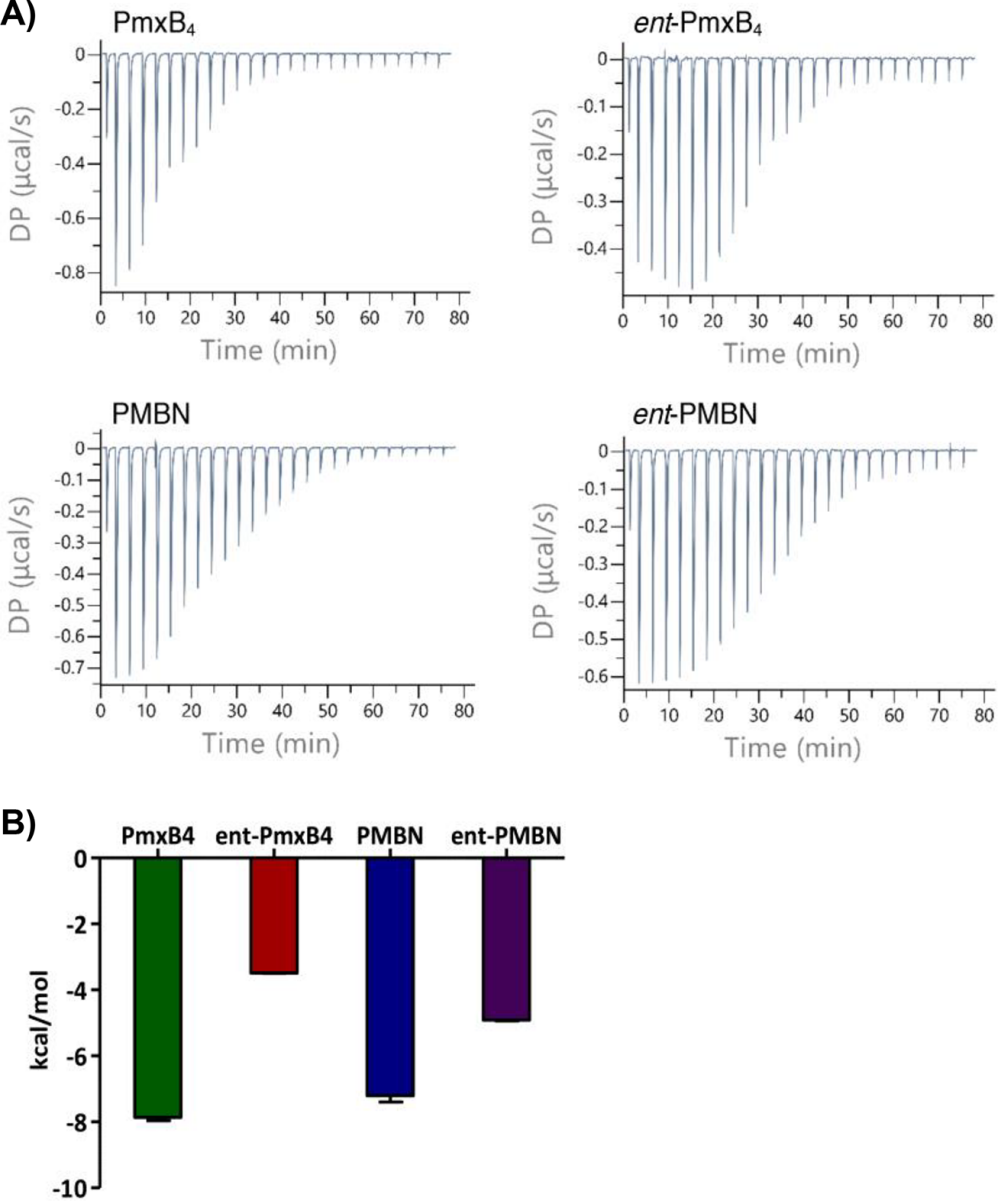
ITC experiments to assess LPS binding by PmxB_4_/ent-PmxB_4_ and PMBN/ent-PMBN. (A) Representative thermograms resulting from titration of the polymyxin peptide indicated (1 mM) into the LPS solution (20 μM). (B) Observed enthalpy change upon titration of peptide into LPS. All experiments were run in triplicate; full data provided in Figures S8–S14 and Table S5.

In summary, we here report a robust synthetic route for the production of polymyxins and have applied it in the preparation of PmxB_4_ along with its novel enantiomer *ent*-PmxB_4_. Antibacterial assays show that, while PmxB_4_ is a potent anti-Gram-negative antibiotic, *ent*-PmxB_4_ is devoid of activity. Moreover, the enantiomer of PMBN, *ent*-PMBN, was also synthesized and found to exhibit a reduced potential, relative to PMBN, to synergize with antibiotics that are typically unable to pass the Gram-negative OM. These findings show that stereochemically defined target recognition is required for both the antibacterial action of full-length polymyxin antibiotics as well as the synergistic activity of the corresponding nonapeptides. Interestingly, despite the absence of inherent antibacterial activity, *ent*-PmxB_4_ does cause OM disruption as evidenced by both its synergistic activity and ability to induce NPN uptake in live bacteria, including a polymyxin-resistant strain. Calorimetric investigations also revealed that, while PmxB_4_ and *ent*-PmxB_4_ both interact with LPS, binding by PmxB_4_ is more efficient. The finding that *ent*-PmxB4 and *ent*-PMBN do interact with LPS, albeit to a lower degree, suggests that, in the absence of precise target recognition, OM binding can still occur, most likely promoted by electrostatic interactions. To conclude, our study is the first to characterize the antibacterial activity of the enantiomer of a full-length polymyxin and clearly reveals the essential stereospecificity of the mechanism of action behind these clinically important antibiotics.

## Methods

### Peptide Synthesis

#### PmxB4 and *ent*-PmxB4

Fmoc-Thr(tBu) was loaded onto chlorotrityl resin via its carboxylic acid. Resin loading was determined to be 0.58 mmol·g^–1^. The linear peptide encompassing the residues p10–p1 was assembled on resin using standard solid phase peptide synthesis (SPPS) conditions using a 4:4:8 ratio of amino acid:BOP:DIPEA relative to the resin. Typical coupling times were 1 h. For coupling of Fmoc-Dab(N3), 2 equiv of amino acid and BOP were used, and coupling time was extended to 4 h. Couplings were done in DMF, and deprotection was achieved using 20% piperidine in DMF (v/v). Amino acids were protected as follows: Boc for Fmoc-Dab, tBu for Fmoc-Thr, and N3 for Fmoc-Dab at p4. For PmxB4, all l-amino acids were used, except for d-Phe at p6. For *ent*-PmxB, all d-amino acids were used, except for l-Phe at p6. After coupling heptanoic acid, the resin was washed well (DMF) and subjected to reduction of the Dab(N3) by a mixture of di-isopropyl amine (DIPA) and dithiothreitol (DTT, racemic variant works well) in dry DMF (3 mL) for 2 h. Reduction was repeated twice. After washings, the peptide was cleaved from the resin by HFIP/DCM (20%, v/v) and cyclized overnight in 100 mL of DMF with DIC (3 equiv) and Oxyma Pure (6 equiv). After completion of cyclization, the mixture was concentrated and subjected to deprotection (TFA/TIPS/H_2_O, 95/2.5/2.5) for 1.5 h. Deprotected peptide was crashed out in ice-cold MTBE and washed with MTBE, followed by lyophilization from *t*-BuOH/H_2_O. Pure material was obtained after RP-HPLC purification (20–50% B gradient).

#### *ent*-PMBN

*ent*-PMBN was synthesized in a similar way as *ent*-PmxB4. After coupling Dab at p3, Boc-d-Thr(tBu) was used as the final amino acid. The rest of the procedure is identical to that of *ent*-PmxB4.

#### PMBN

PMBN was synthesized from polymyxin B with slight modifications of previously described methods^37^ as follows: Polymyxin B sulfate (commercially available mixture of isomers, Combi-Blocks, San Diego, USA, 4.0 g, 2.8 mmol) was dissolved in demiwater (120 mL). Dithiothreitol (DTT, 106 mg, 0.69 mmol) was added to the solution, followed by ficin (1.06 g, ∼44 μmol). Enzymatic digestion was run at 37 °C under N_2_ atmosphere overnight. Reaction progress was monitored by LC-MS. Additional dithiothreitol (16 mg, 63 μmol) and ficin (160 mg) were added, and digestion was run overnight once more. Once complete, the mixture was heated to reflux for 20 min, followed by filtration of the precipitate. The pH of the filtrate was adjusted to pH 2, and the sample was extracted with *n*-butanol (4 × 35 mL). The aqueous layer was freeze-dried from *t*-BuOH/H_2_O. RP-HPLC was used to obtain pure PMBN (0–40% gradient).

### MIC Experiments

MICs of compounds were determined following CLSI guidelines using the microbroth dilution method in polypropylene plates. Selected Gram-negative bacterial strains (see Table S2 for strain sources) were taken from glycerol stocks and grown overnight on blood agar. Individual colonies were selected and grown in Tryptic Soy Broth (TSB) to an OD_600_ of 0.5. Compounds were diluted in Mueller Hinton Broth (MHB) supplemented with Mg^2+^ and Ca^2+^ at final concentrations of 12 and 20 mg/L, respectively (CAMHB). 50 μL was used for each well, and compounds were assessed in triplicates. Bacterial culture was diluted in CAMHB to 10^6^ CFU/mL, yielding 5 × 10^5^ CFU/mL upon addition of 50 μL of culture to 50 μL of compound solution. Positive and negative (sterility) controls were present on each plate. Plates were sealed with a semipermeable membrane and incubated for 22 h (*A. baumannii*) or 18 h (other strains) under shaking at 37 °C. MICs are defined as the lowest concentration of compound that visibly inhibits bacterial growth.

### Checkerboard Assays

Selected Gram-negative bacterial strains were taken from glycerol stocks and grown overnight on blood agar. A single colony was inoculated in TSB broth at 37 °C until an optical density of 0.5 at 600 nm (OD_600_) was reached. The bacterial suspension was diluted in freshly prepared CAMHB to 10^6^ CFU/mL. Compounds were diluted along the plate, using 25 μL of CAMHB for each dilution. Antibiotic concentrations were prepared separately in a multiwell reservoir, including a control without antibiotic. Of each antibiotic stock, 25 μL was added to three columns to obtain triplicates for every condition. To each 50 μL containing well was then added 50 μL of the 10^6^ CFU/mL bacterial suspension, and the plates were sealed with breathable seals. After incubation for 18 h at 37 °C while shaking, the seals were removed and the density of the bacterial suspensions was measured at 600 nm (OD_600_) using a Tecan Spark plate reader. The OD_600_ values were transformed to a 2D gradient with the negative control as the minimum and the positive control as the maximum. The FICI was calculated using the following equation:
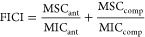
with MSC_ant_ and MSC_comp_ indicating the optimal synergistic concentration of antibiotic and compound and MIC_ant_ and MIC_comp_, the corresponding MICs of the individual compounds. An FICI ≤ 0.5 indicates synergy.^48^

### NPN Assay

Bacteria were taken from glycerol stocks and grown in TSB overnight. Cultures were diluted either 50 or 100 times in Lysogeny Broth (LB) and grown at 37 °C to an OD_600_ of 0.5. Test compounds were prepared in HEPES buffer (5 mM, pH 7.2 + 20 mM glucose) and serially diluted (25 μL per well) on 1/2 the area of black 96-well plates with a transparent bottom. *N*-Phenyl-1-naphthylamine (NPN) was dissolved in acetone (0.5 mM) and diluted to 40 μM in HEPES buffer (5 mM, pH 7.2 + 20 mM glucose). 25 μL of the NPN solution was added to each well. The bacterial suspension was spin down (1000*g*, 10 min), and the remaining cell pellet was resuspended in half the original volume of HEPES buffer (5 mM, pH 7.2 + 20 mM glucose). 50 μL of bacterial suspension was added to each well. NPN fluorescence was measured after 1 h of incubation at RT with λ_ex_ 355 ± 20 nm and λ_em_ 420 ± 20 nm. The measured fluorescence signal was corrected for NPN fluorescence in the absence of compounds (bacteria in buffer + NPN) and presented as relative fluorescence units (RFUs).

### Isothermal Titration Calorimetry (ITC)

Stock solutions of LPS (10 mg/mL, 0.5 mM) were prepared in 20 mM HEPES at pH 7 by suspension, sonication, and temperature cycling between 4 and 60 °C and stored overnight prior to use. LPS from *E. coli* (O55:B5) was used. Lipopeptide solutions (1 mM in 20 mM HEPES, pH 7) were titrated into a suspension of LPS (0.020 mM) in the same buffer. Control titrations included the titration of peptide into buffer and buffer into the LPS suspension to determine the corresponding heat of dilution. All binding experiments were performed using a MicroCal PEAQ-ITC Automated microcalorimeter (Malvern Panalytical Ltd., Malvern, UK). The samples were equilibrated to 37 °C prior to measurement. The titrations were conducted at 37 °C under constant stirring at 750 rpm. Each experiment consisted of an initial injection of 0.3 μL followed by 25 separate injections of 1.5 μL into the sample cell of 200 μL. The time between each injection was 180 s, and the measurements were performed with the reference power set at 5 μcal s^–1^ and the feedback mode set at “low”. For all calculations performed, an estimated molecular weight of 20 kDa for LPS was applied. The calorimetric data thus obtained were analyzed using MicroCal PEAQ-ITC Analysis Software Version 1.20 (Malvern Panalytical Ltd., Malvern, UK), where the integrated heat signal is corrected for the heat of dilution of the titrant.

## Supporting Information Available

Abbreviations, reagents and general procedures, HRMS data, HPLC traces, and 1D and 2D NMR spectra for all peptides synthesized, information on bacterial strains, additional MIC analyses and FICI calculations, and ITC thermograms for all binding experiments performed (PDF)
