# Supplementary material for: Polymyxin Stereochemistry and Its Role in Antibacterial Activity and Outer Membrane Disruption
Source: ACS Infect Dis. 2022 Nov 7;8(12):2396–404. doi: 10.1021/acsinfecdis.2c00307 (PMC9745799; doi:10.1021/acsinfecdis.2c00307)
Supplement: Supplementary file 1 — id2c00307_si_001.pdf [file id2c00307_si_001.pdf]

Supporting information for:

Polymyxin Stereochemistry and its Role in Antibacterial Activity and Outer Membrane Disruption

Cornelis J. Slingerland[1], Ioli Kotsogianni[1], Charlotte M.J. Wesseling[1], Nathaniel I. Martin[1,*]

[1]Biological Chemistry Group, Institute of Biology Leiden, Leiden University, Sylviusweg 72, 2333 BE Leiden, The Netherlands. *n.i.martin@biology.leidenuniv.nl

Table of contents

S1	Abbreviations
S2	Reagents and General Procedures
S3-4	HRMS and HPLC analyses
S5-6	NMR analyses
S7	HPLC/MS analysis of commercially obtained polymyxin B
S8	Information on used bacterial strains
S9	Additional MIC analyses and values for FICI calculations
S10 – S13	Isothermal calorimetry data

Abbreviations:

^{t}BuOH: tert-butanol; BOP: (benzotriazole-1-yloxy)tris-(dimethylamino)phosphonium hexafluorophosphate; CTC: 2-chloro tritylchloride; Dab: 2,4-Diaminobutyric acid; DIPEA: *N,N*-diisopropylethylamine; DIPA: *N,N*-di-isopropyl amine; DIC: *N,N'*-Diisopropylcarbodiimide; FICI: fractional inhibitory concentration index; mcr: mobile colistin resistance; OM: outer membrane; Oxyma pure: Ethyl cyano(hydroxyimino)acetate; PMBN: polymyxin B nonapeptide; PmxB: Polymyxin B; TIPS: tri-isopropyl silane.

Reagents and General Procedures

General Procedures

All reagents employed were of American Chemical Society (ACS) grade or finer and were used without further purification unless otherwise stated. Polymyxin B sulphate was obtained as a mixture of isomers (Combi-Blocks, San Diego, USA). An HPLC trace of the mixture, including identification of the main species is shown in Figure S7. For compound characterization HRMS analysis was performed on a Shimadzu Nexera X2 UHPLC system with a Waters Acquity HSS C18 column (2.1 × 100 mm, 1.8 µm) at 30 °C and equipped with a diode array detector. The following solvent system, at a flow rate of 0.5 mL/min, was used: solvent A, 0.1 % formic acid in water; solvent B, 0.1 % formic acid in acetonitrile. Gradient elution was as follows: 95:5 (A/B) for 1 min, 95:5 to 15:85 (A/B) over 6 min, 15:85 to 0:100 (A/B) over 1 min, 0:100 (A/B) for 3 min, then reversion back to 95:5 (A/B) for 3 min. This system was connected to a Shimadzu 9030 QTOF mass spectrometer (ESI ionisation) calibrated internally with Agilent's API-TOF reference mass solution kit (5.0 mM purine, 100.0 mM ammonium trifluoroacetate and 2.5 mM hexakis(1H,1H,3H-tetrafluoropropoxy)phosphazine) diluted to achieve a mass count of 10000.

Purity of the peptides was confirmed to be ≥ 95% by analytical RP-HPLC using a Shimadzu Prominence-i LC-2030 system with a Dr. Maisch ReproSil Gold 120 C18 column (4.6 × 250 mm, 5 µm) at 30 °C and equipped with a UV detector monitoring at 214 nm. The following solvent system, at a flow rate of 1 mL/min, was used: solvent A, 0.1 % TFA in water/acetonitrile, 95/5; solvent B, 0.1 % TFA in water/acetonitrile, 5/95. Gradient elution was as follows: 100:0 (A/B) for 3 min, 100:0 to 0:100 (A/B) over 47 min, 0:100 (A/B) for 4 min, then reversion back to 100:0 (A/B) over 1 min, 100:0 (A/B) for 5 min.

The compounds were purified via preparative HPLC using a BESTA-Technik system with a Dr. Maisch Reprosil Gold 120 C18 column (25 × 250 mm, 10 µm) and equipped with a ECOM Flash UV detector monitoring at 214 nm. The following solvent system, at a flow rate of 12 mL/min, was used: solvent A, 0.1 % TFA in water/acetonitrile 95/5; solvent B, 0.1 % TFA in water/acetonitrile 5/95. Gradient elution was as follows: for PMBN and *ent*-PMBN: 100:0 (A:B) for 3 min, 100:0 to 90:10 (A:B) over 3 min, 90:10 to 60:40 (A:B) over 41 min, 60:40 to 0:100 (A:B) over 1 min, 0:100 (A:B) for 4 min, then reversion back to 100:0 (A:B) over 1 min, 100:0 (A:B) for 2 min.

For PmxB4 and ent-Pmx4: 100:0 (A:B) for 3 min, 100:0 to 85:15 (A/B) over 4 min, 85:15 to 55:45 (A:B) over 41 min, 55:45 to 0:100 (A:B) over 1 min, 0:100 (A:B) for 4 min, then reversion back to 100:0 (A:B) over 1 min, 100:0 (A:B) for 2 min.

HRMS analysis

Table S1. HRMS data for polymyxin analogues

**Compound**	**Composition**	**Calculated mass**	**[M+H]$^+$**	**[M+2H]/2**	**Found mass**
PmxB4	$C_{54}H_{94}N_{16}O_{13}$	1174.7186	1175.7266	588.3673	588.3675
ent-PmxB4	$C_{54}H_{94}N_{16}O_{13}$	1174.7186	1175.7266	588.3673	588.3674
PMBN	$C_{43}H_{74}N_{14}O_{11}$	962.5661	963.5741	482.2911	482.2909
ent-PMBN	$C_{43}H_{74}N_{14}O_{11}$	962.5661	963.5741	482.2911	482.2907

HPLC analysis

PmxB4

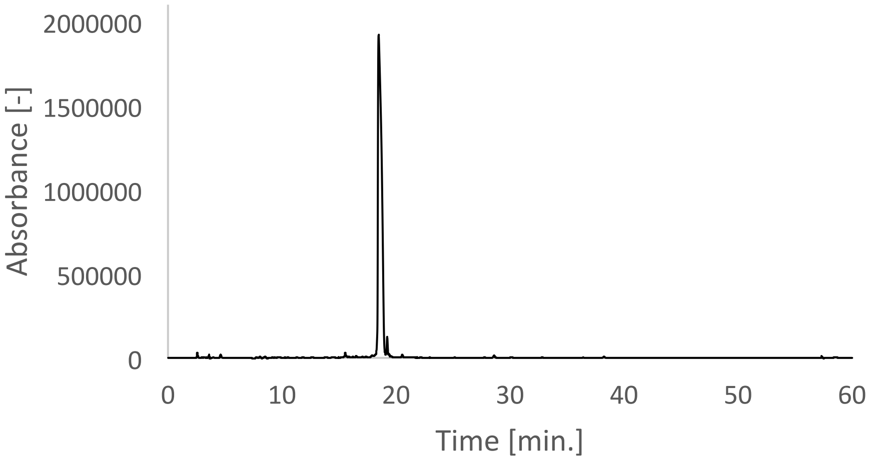

ent-PmxB4

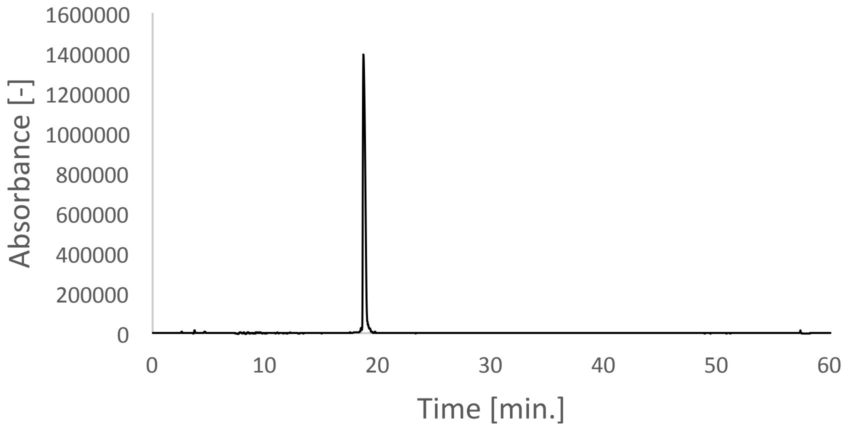

Figure S1A. Analytical HPLC traces for enantiomers PmxB4 and *ent*-PmxB4.

PMBN

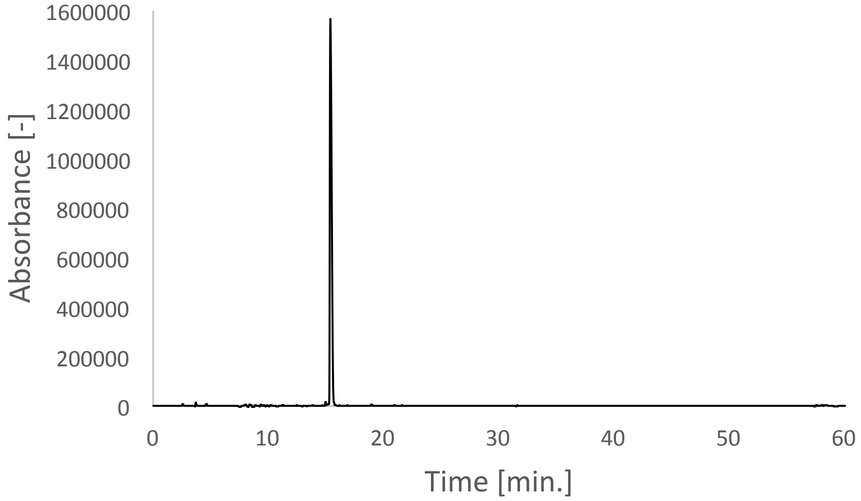

ent-PMBN

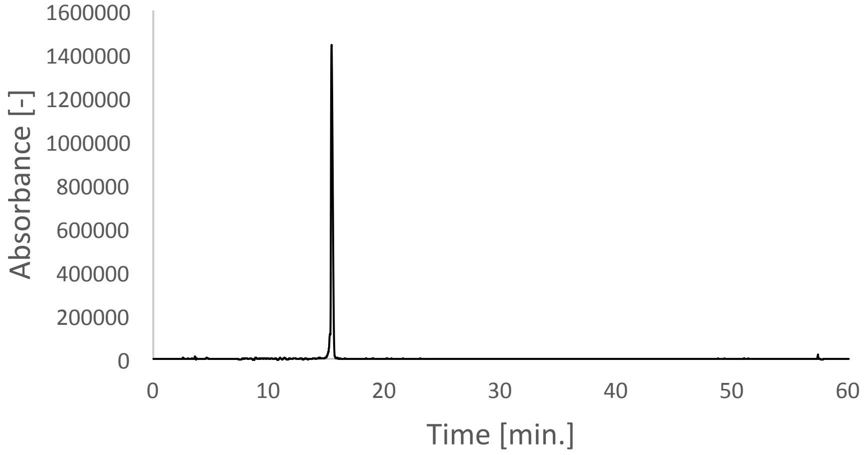

Figure S1B. Analytical HPLC traces for enantiomers PMBN and *ent*-PMBN

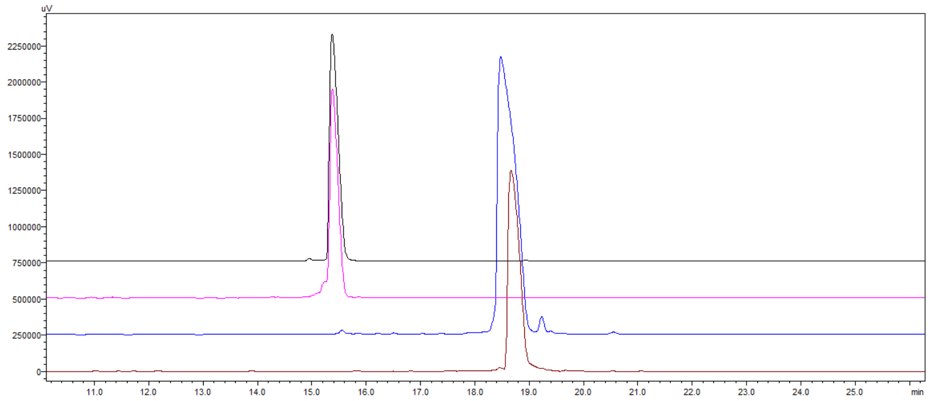

Figure S2. Overlay of analytical HPLC traces of PmxB4 (blue), *ent*-PmxB4 (brown), PMBN (black) and *ent*-PMBN (purple) indicating enantiomerism between PmxB4/*ent*-PmxB4 and PMBN/*ent*-PMBN.

NMR analysis

PmxB4, *ent*-PmxB4, PMBN and *ent*-PMBN were analyzed by both 1D and 2D NMR. Samples were dissolved in 90% H_2O, 10% D_2O, with the addition of TFA to a final concentration of 0.1%. NMR spectra were recorded at 850 MHz, at ambient temperature. Watergate water suppression was used. Samples of PmxB4 and *ent*-PmxB4 were compared to PmxB1, as isolated from a commercially available polymyxin mixture.

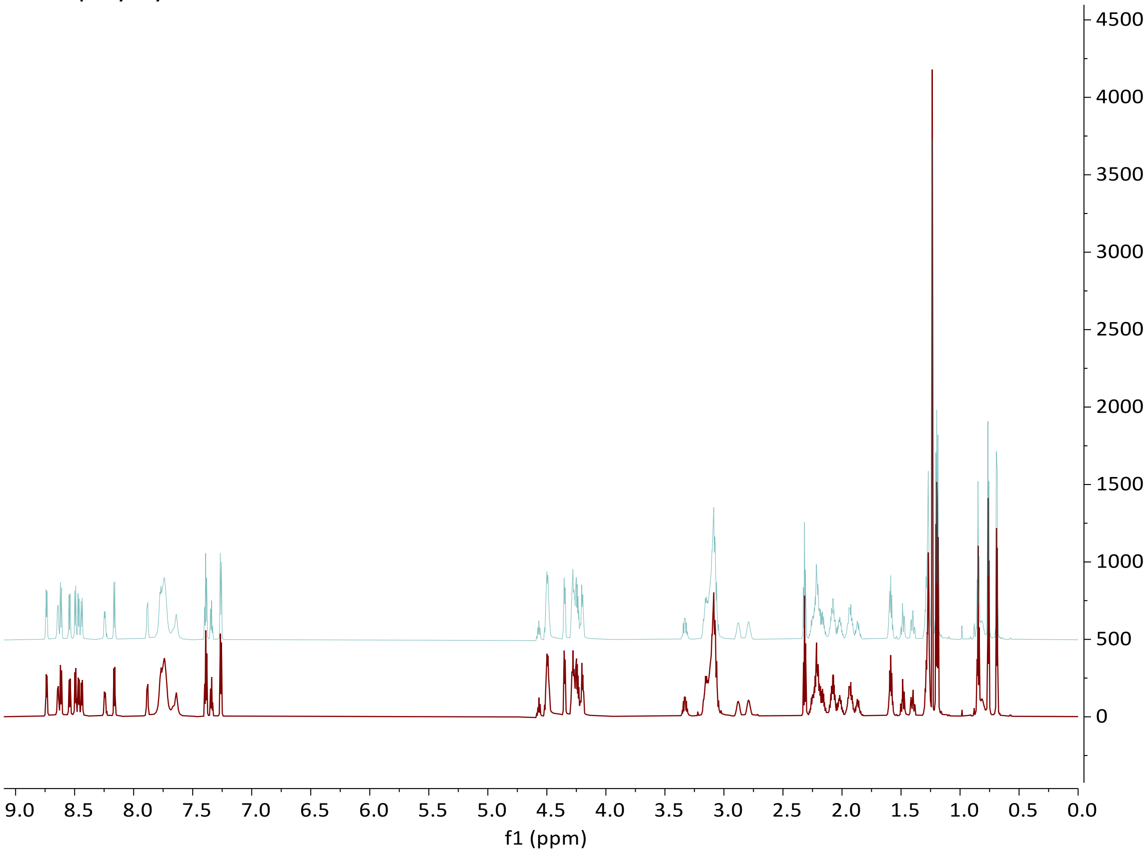

Figure S3. ^{1}H NMR comparison between *ent*-PmxB4 (top, blue) and PmxB4 (bottom, red) showing identity between the two samples.

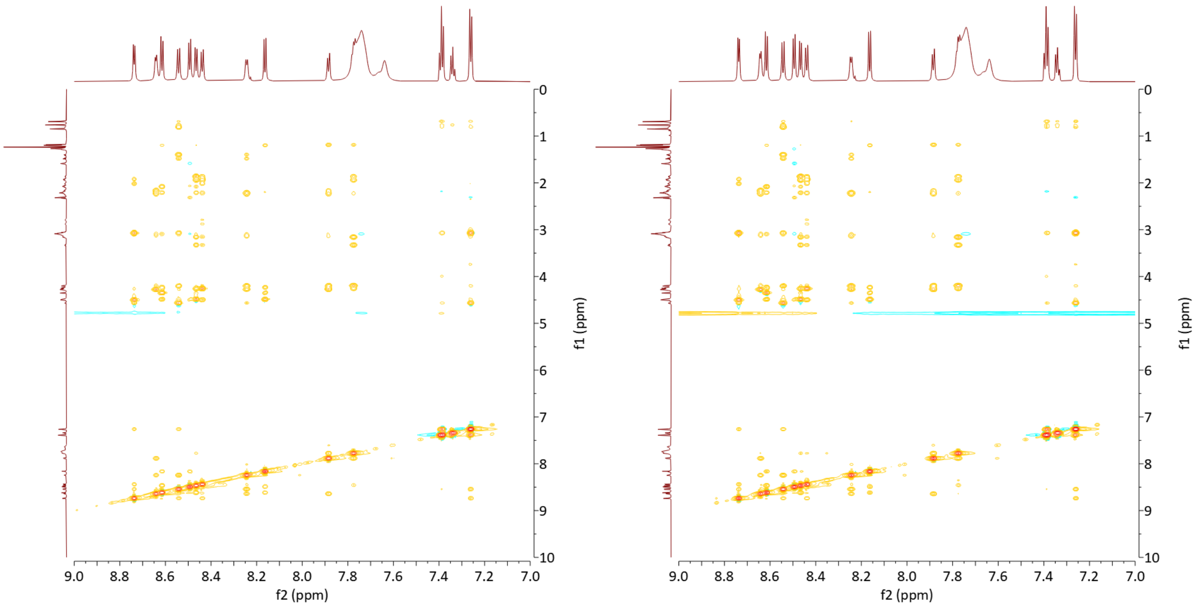

Figure S4. NOESY comparison between *ent*-PmxB4 (left) and PmxB4 (right) showing identity between the two samples.

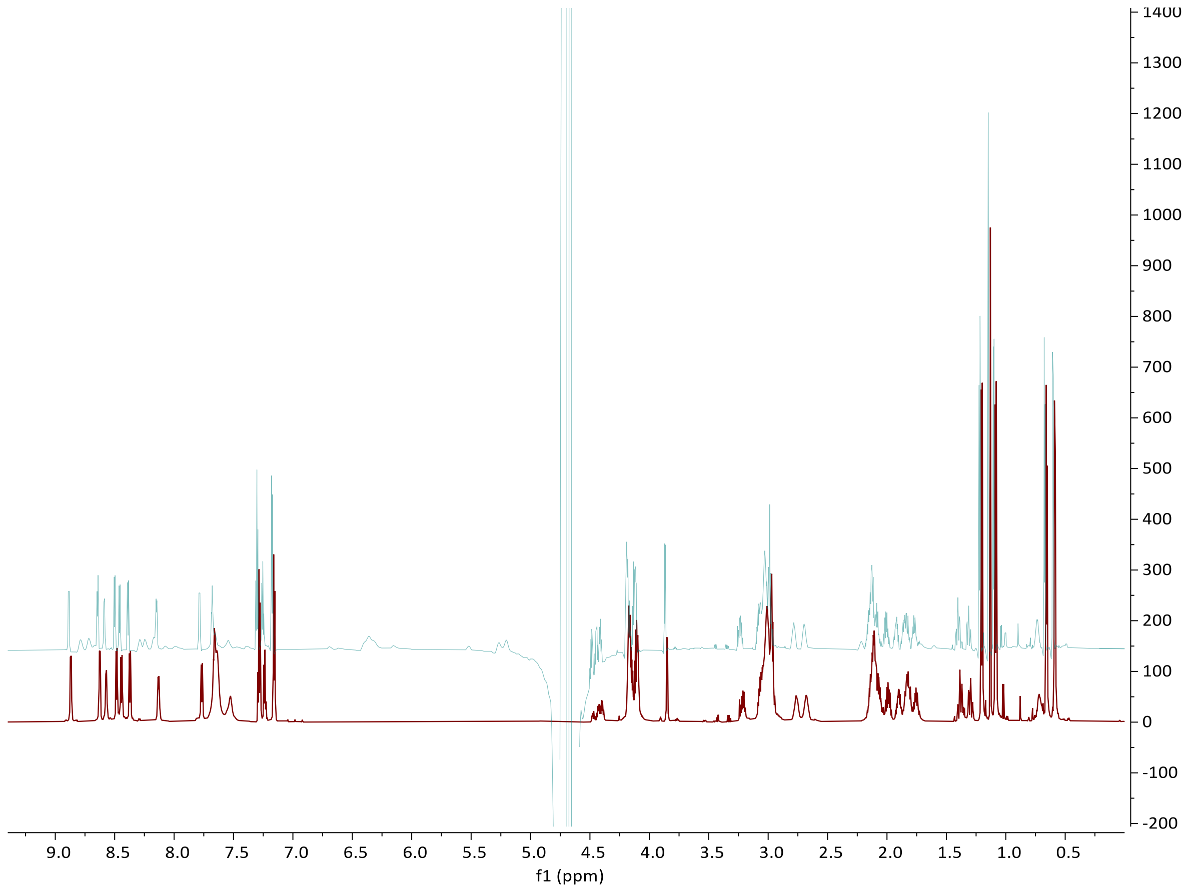

Figure S5. ^{1}H NMR comparison between *ent*-PMBN (top, blue) and PMBN (bottom, red) showing identity between the two samples.

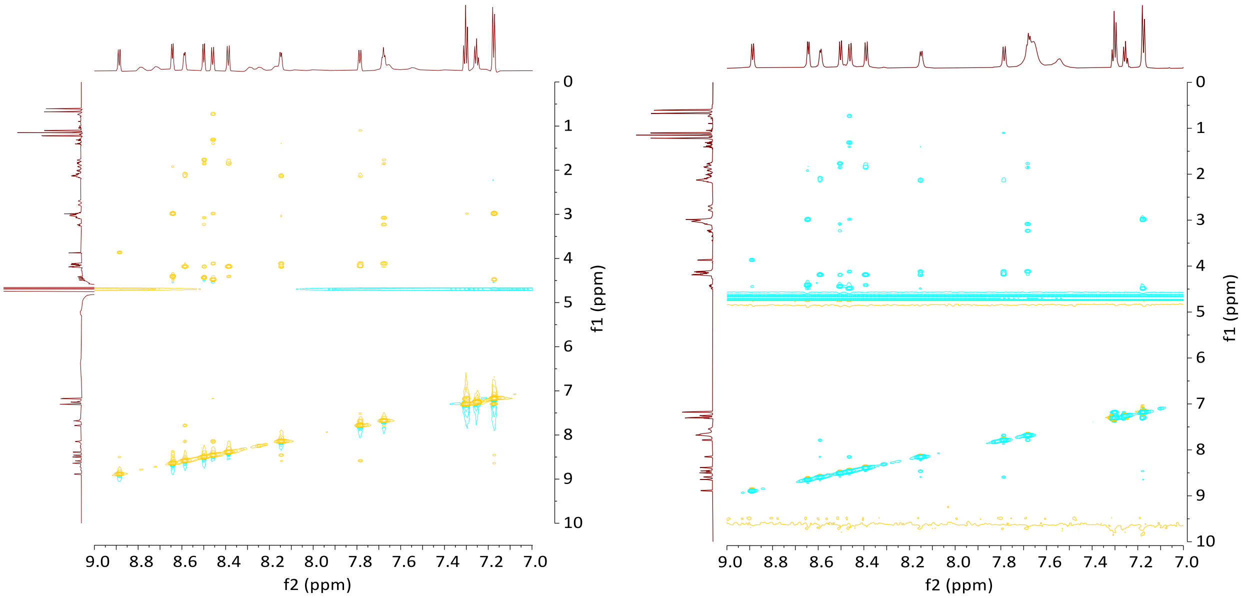

Figure S6. NOESY comparison between *ent*-PmxB4 (left) and PmxB4 (right) showing identity between the two samples.

HPLC/MS analysis on polymyxin B from commercial source

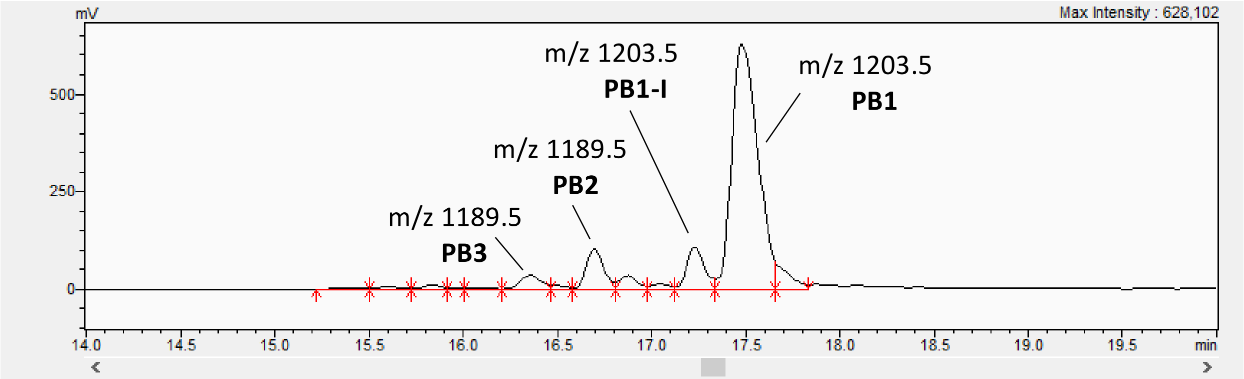

Results View - Peak Table

Peak Table | Compound | Group | Calibration Curve

Peak#	Peak Start	Peak End	Ret. Time	Area	Height	Area%
1	15.225	15.500	15.408	26317	2783	0.311
2	15.500	15.725	15.603	43932	6356	0.520
3	15.725	15.917	15.831	65696	10442	0.777
4	15.917	16.008	15.929	13571	2635	0.161
5	16.008	16.208	16.021	17048	2104	0.202
6	16.208	16.467	16.359	263773	35132	3.120
7	16.467	16.583	16.479	43839	8777	0.519
8	16.583	16.808	16.697	651197	101335	7.703
9	16.808	16.975	16.872	211102	33582	2.497
10	16.975	17.125	17.046	82063	12018	0.971
11	17.125	17.333	17.230	687153	104923	8.128
12	17.333	17.658	17.481	6023843	626739	71.254
13	17.658	17.833	17.671	324524	62415	3.839
Total				8454060	1009242	100.000

Figure S7. Analysis of the commercially obtained Polymyxin B, typically supplied as mixture of isomers. Observed m/z values for main polymyxin B species are indicated in the chromatogram. PB: polymyxin B; PB1-I: polymyxin B_1 with isoleucine at position 7.

Bacterial strains

Table S2. Information on strain identity and origin.

Strain	Strain designator	Resistance	Source
E. coli	ATCC 25922		ATCC collection
	BW25113		Utrecht University Medical Center (UMC) – Microbiology Department. Heidelberglaan 100, 3584 CX Utrecht, The Netherlands
	mcr-1	mcr-1	Clinical isolate, Utrecht University Medical Center (UMC) – Microbiology Department. Heidelberglaan 100, 3584 CX Utrecht, The Netherlands
	JW3594 (ΔwaaD)		E. Coli BW25113 ΔrfaD. Keio knock-out collection
	JW3596 (ΔwaaC)		E. Coli BW25113 ΔrfaC. Keio knock-out collection
K. pneumoniae	ATCC 13883		ATCC collection
A. baumannii	ATCC 19606		ATCC collection
P. aeruginosa	ATCC 27853		ATCC collection
	NRZ03961	IMP-1	National reference laboratory for multidrug-resistant gram-negative bacteria, department for medical microbiology, Ruhr-University Bochum, Universitaetsstr. 150, 44801 Bochum, Germany.
	ATCC 10145		ATCC collection
	2018-007	IMP-7	The Dutch national institute for public health and the environment (RIVM), Antonie van Leeuwenhoeklaan 9, 3721 MA Bilthoven, The Netherlands

MICs in presence of PMBN/SPR741

Table S3. MIC values for *ent*-PmxB4, in presence of either PMBN or SPR741.

		+ PMBN (8 ug/mL)	+ SPR741 (8 ug/mL)
E. coli	**ATCC 25922**	128	128
K. pneumoniae	**ATCC 13883**	>128	>128
A. baumannii	**ATCC 19606**	>128	>128
P. aeruginosa	**ATCC 10145**	>128	>128

FICI values

Table S4. MIC values of rifampicin, PmxB4, *ent*-PmxB4, PMBN and *ent*-PMBN, used for calculation of FICI values shown in Figure 2.

		MIC alone (µg/mL)	MIC in combination (µg/mL)		MIC alone (µg/mL)	MIC in combination (µg/mL)	FICI
**E. coli ATCC 25922**	Rifampicin	8	0.5	PmxB4	1	1	**> 0.5**
	Rifampicin	8	0.1	*ent*-PmxB4	128	8	**0.08**
	Rifampicin	8	0.1	PMBN	≥256	2	**≤ 0.02**
	Rifampicin	8	2	*ent*-PMBN	≥256	32	**≤ 0.38**
**E. coli mcr-1**	Rifampicin	8	0.3	PmxB4	8	1	**0.16**
	Rifampicin	8	1	*ent*-PmxB4	128	8	**0.19**
	Rifampicin	8	1	PMBN	≥256	4	**≤ 0.14**
	Rifampicin	8	2	*ent*-PMBN	≥256	32	**≤ 0.38**

Isothermal calorimetry (ITC) data

Control titrations
1. Buffer into LPS

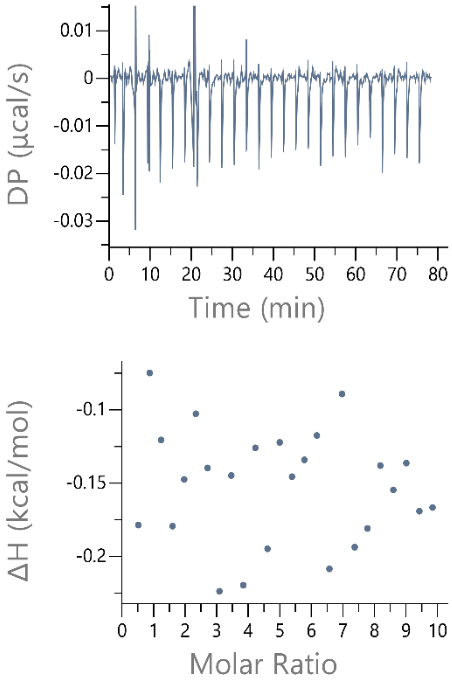

Figure S8. Control titration into LPS. Buffer was titrated into LPS (20 µM, from *E. Coli* O55:B5).

Control titrations
2. Lipopeptides into buffer

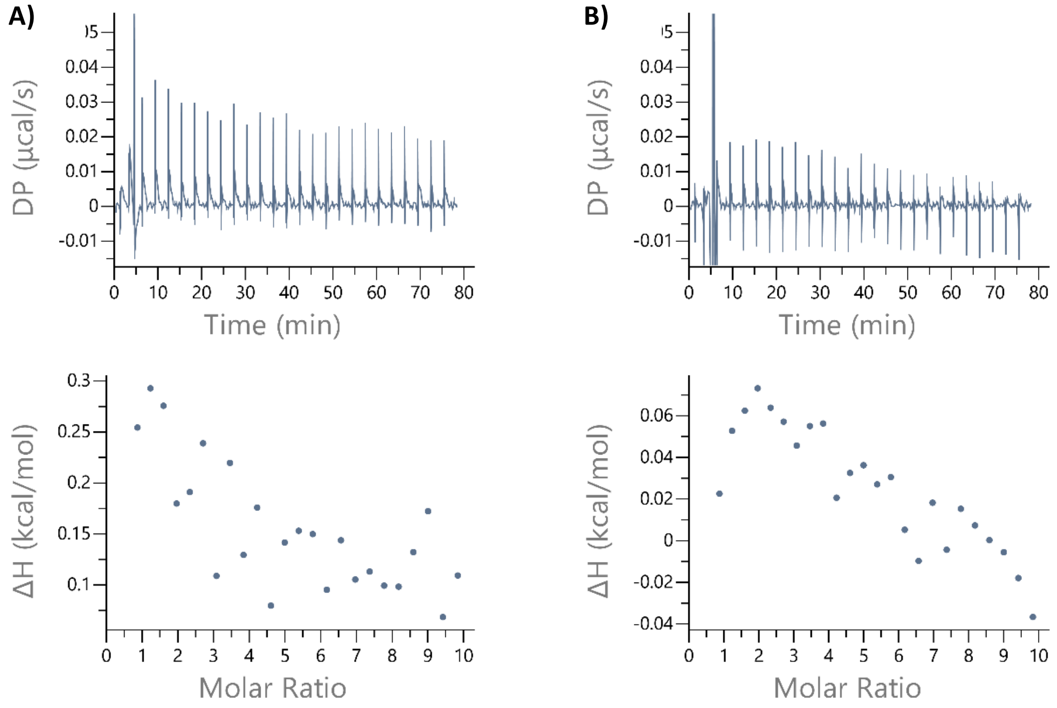

Figure S9. Control titrations into buffer. **A)** PmxB4 (1 mM) was titrated into buffer. **B)** *ent*-PmxB4 (1 mM) was titrated into buffer.

Control titrations
3. Nonapeptides into buffer

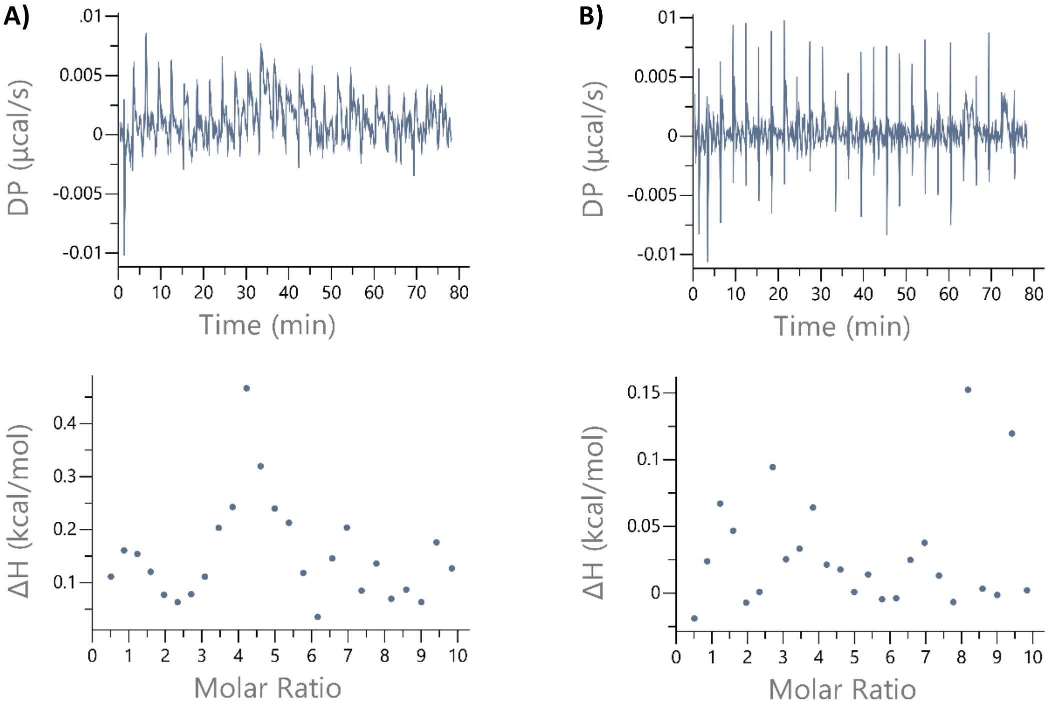

Figure S10. Control titrations into buffer. **A)** PMBN (1 mM) was titrated into buffer. **B)** *ent*-PMBN (1 mM) was titrated into buffer.

Titrations of compounds into LPS (triplicates).
1. Titration of PmxB4 (1 mM) into LPS (20 uM).

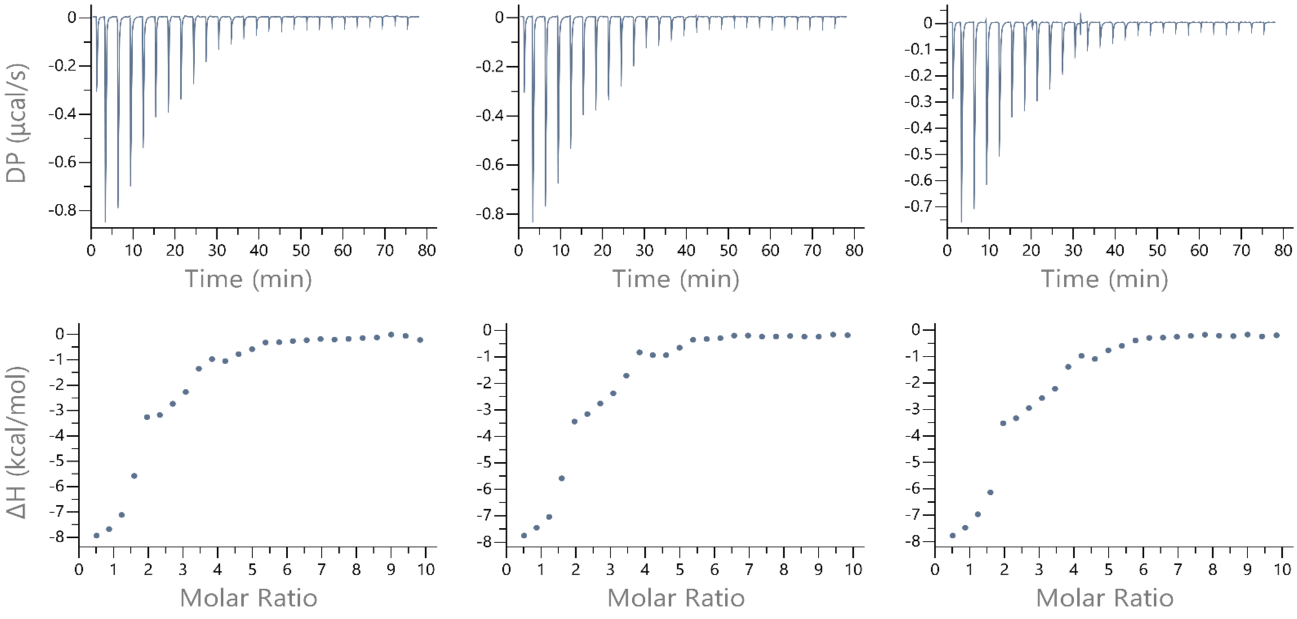

Figure S11. Triplicate experiment of titration of PmxB4 into LPS (20 µM).

Titrations of compounds into LPS (triplicates).
2. Titration of ent-PmxB4 (1 mM) into LPS (20 uM).

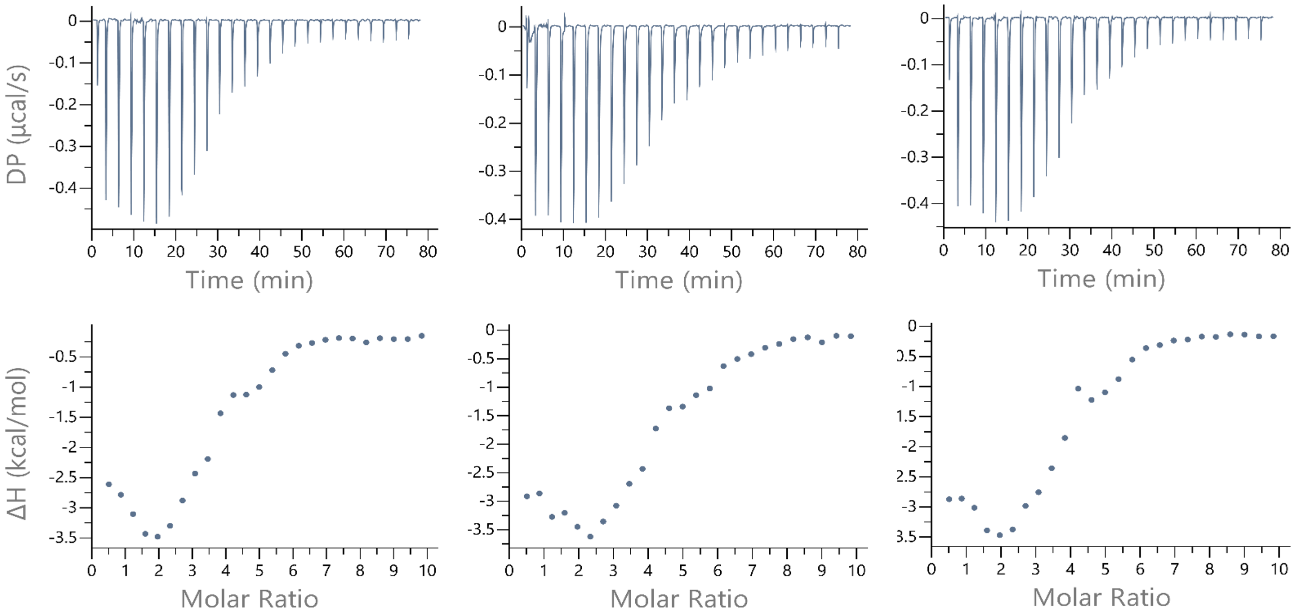

Figure S12. Triplicate experiment of titration of *ent*-PmxB4 into LPS (20 µM).

Titrations of compounds into LPS (triplicates).
3. Titration of PMBN (1 mM) into LPS (20 uM).

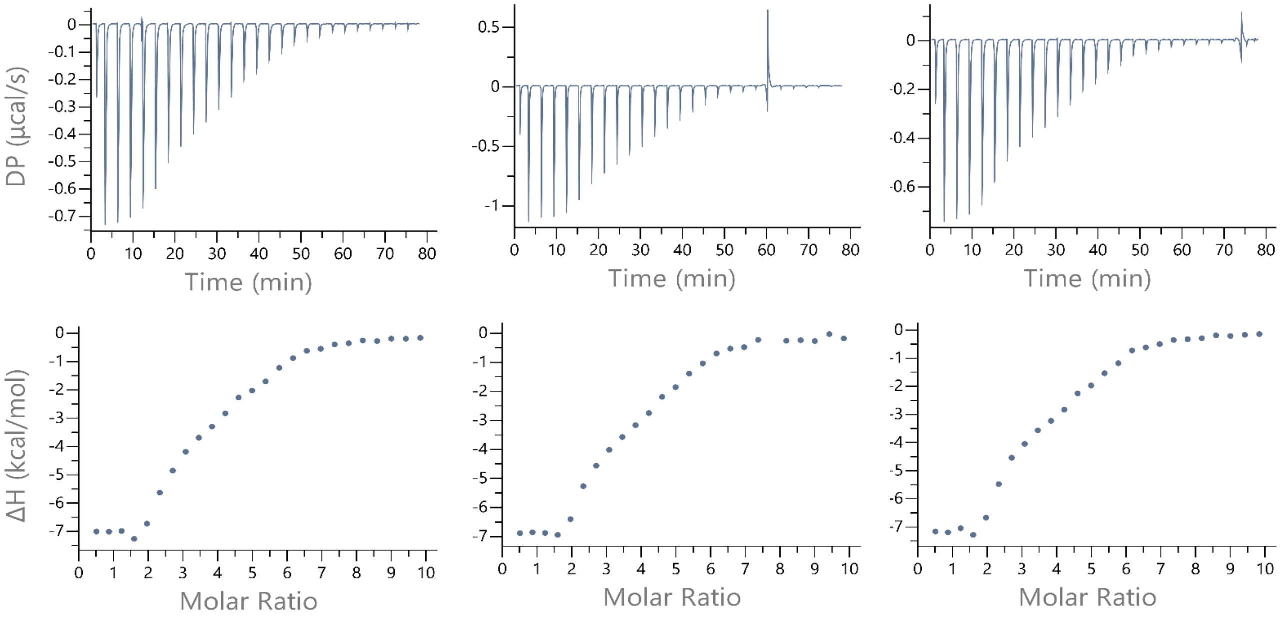

Figure S13. Triplicate experiment of titration of PMBN into LPS (20 µM).

Titrations of compounds into LPS (triplicates).
4. Titration of ent-PMBN (1 mM) into LPS (20 uM).

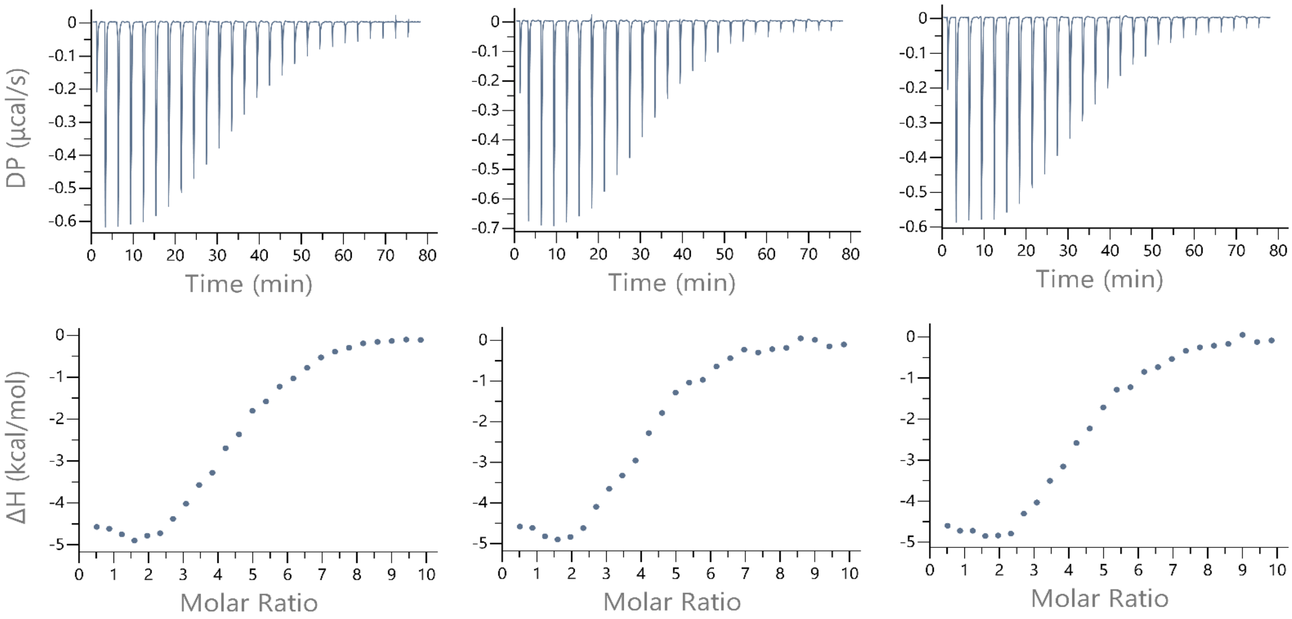

Figure S14. Triplicate experiment of titration of *ent*-PMBN into LPS (20 µM).

Table S5. Thermodynamic analysis from the titration of compounds (1 mM) in LPS (20µM) at 37°C. Mean values and standard deviation from three experiments are shown.

	**ΔΔH[a] (kcal/mol)**	**Fitting Model**	**Fitted Parameters for Single Site binding**			
			**ΔH (kcal/mol)**	**ΔG (kcal/mol)**	**K_D (µM)**	**Peptide/LPS[b]**
PmxB4	-7.86 ± 0.10	ND[c]	ND	ND	ND	ND
ent- PmxB4	-3.485 ± 0.015	ND[c]	ND	ND	ND	ND
PMBN	-7.20 ± 0.19	One-site	-8.4 ± 0.25	-7.24 ± 0.03	8.0 ± 0.4	3.5
ent- PMBN	-4.91 ± 0.03	One-site	-5.2 ± 0.03	-7.69 ± 0.06	3.9 ± 0.4	4.3

[a] Maximum enthalpy observed after subtraction of dilution control. Global analysis. [b]Molar ratio of peptide to LPS preparation assuming 20 kDa molecular weight. [c]ND: Not determined. No model was able to fit the global titration curve. Attempts included fitting in the following binding models: one set of sites, two set of sites, sequential binding sites with 2,3 and 4 sites.
